# Canine Periodontal Disease in an Urban Clinical Setting and Its Relationship with Host Factors and the Subgingival Bacterial Profile in Ecuador

**DOI:** 10.3390/ani16111630

**Published:** 2026-05-27

**Authors:** David Martínez-Matamoros, Miriam Sánchez-Vivanco, Jessica Valdivieso-Tituana, Orlando Meneses-Quelal

**Affiliations:** 1 Carrera de Medicina Veterinaria, Universidad Politécnica Estatal del Carchi, Carchi 100105, Ecuador; davidf.martinez@upec.edu.ec; 2 Facultad Agropecuaria y de Recursos Naturales Renovables, Universidad Nacional de Loja, Loja 1101608, Ecuador; miriam.sanchez@unl.edu.ec (M.S.-V.); jessica.valdivieso@unl.edu.ec (J.V.-T.); 3 Carrera de Alimentos, Universidad Politécnica Estatal del Carchi, Carchi 100105, Ecuador

**Keywords:** canine periodontal disease, subgingival microbiota, host factors

## Abstract

Canine periodontal disease is a highly prevalent chronic inflammatory condition with a multifactorial etiology, influenced by host factors and complex subgingival bacterial communities; however, evidence from populations in underrepresented regions remains limited. The objective of this study was to evaluate the association between host factors, including age, diet, and cranial morphology, and the presence and severity of canine periodontal disease in dogs treated in an urban clinical setting in Ecuador, as well as to characterize the subgingival bacterial profile recoverable by culture-based methods. A cross-sectional, analytical, observational study was conducted on 100 dogs treated at veterinary clinics in Loja, southern Ecuador. Periodontal status was classified according to AVDC criteria, defining the outcomes as the presence of canine periodontal disease (stages 1–4 vs. 0) and advanced periodontitis (stages 2–4 vs. 0–1). Subgingival samples were collected using sterile paper points and processed under aerobic and anaerobic conditions, with analyses performed individually. Canine periodontal disease was present in 68.0% of dogs, with 37.0% in advanced stages. Age was the only factor independently associated with both the presence (OR = 1.18; 95% CI: 1.02–1.36; *p* = 0.021) and severity (OR = 1.22; 95% CI: 1.05–1.41; *p* = 0.009), while diet, sex, and cranial morphology showed no significant associations (*p* > 0.05). The bacterial profile was polymicrobial (3.86 positive isolates per individual), and no taxon showed a significant association after FDR correction. Taken together, these results support a multifactorial and polymicrobial model, highlighting age as the main associated factor and emphasizing the need for molecular approaches in future studies.

## Simple Summary

Canine periodontal disease is one of the most common health problems in dogs and can affect their quality of life and overall health. This study aimed to better understand the types of bacteria present below the gum line and to evaluate whether factors such as diet, skull shape, and severity of gum inflammation are associated with more severe disease. To do this, samples were collected from dogs treated in veterinary clinics in southern Ecuador and analyzed under controlled laboratory conditions. The results showed that the oral environment is highly complex, with multiple types of bacteria coexisting in each animal. Although some patterns were observed, factors such as diet type and skull shape did not consistently predict severe microbial imbalance when analyzed individually. These findings suggest that canine periodontal disease in dogs is not caused by a single factor but rather by the interaction of multiple biological and environmental conditions. Understanding this complexity is important because it can help veterinarians develop more accurate diagnostic approaches and improve prevention strategies, ultimately contributing to better animal health and welfare.

## 1. Introduction

Canine periodontal disease is the most prevalent chronic inflammatory condition in veterinary practice worldwide, affecting more than 80% of dogs over two years of age [1]. Far from being a pathological process confined to the oral cavity, PD is currently conceptualized from the One Health perspective as a low-grade endotoxemia that induces chronic systemic inflammation. Ulceration of the gingival sulcus epithelium and the resulting transient bacteremia facilitate the hematogenous dissemination of live immunogens and pathogens, establishing undeniable epidemiological correlations with distant organ dysfunctions, including myxomatous endocarditis, interstitial nephritis, and premature hepatic senescence [2].

Despite its clinical ubiquity, the etiology of anatomical attachment loss (apical migration of the junctional epithelium and alveolar bone resorption) has undergone a radical paradigm shift in the last decade. The classic infectious model, focused on absolute bacterial load or specific pathogens, has been replaced by the Polymicrobial Synergy and Dysbiosis (PSD) Model [3]. In this theoretical framework, periodontal inflammation is not the result of exogenous invasion, but rather the product of a breakdown in endogenous ecological homeostasis. The canine subgingival biofilm harbors a highly structured ecosystem of more than 300 bacterial phylotypes [4]. Under conditions of clinical eubiosis, this niche is dominated by primary aerobic and facultative anaerobic colonizers, predominantly asaccharolytic Gram-positive bacteria (*Staphylococcus* spp., *Streptococcus* spp., and *Actinomyces* spp. in early stages), which maintain a symbiotic relationship with the host [5].

However, the evolutionary transition to destructive periodontitis (American Veterinary Dental College, AVDC, stages 3 and 4) is catalyzed by an environmental disturbance that alters the thermodynamic equilibrium of the gingival sulcus. The theory known as the “Ecological Plaque Hypothesis” postulates that substrate stasis and the lack of mechanical disruption allow for the three-dimensional stratification of the biofilm [6]. As the biofilm matures, peripheral oxygen consumption generates an extreme hypoxic gradient in the deeper layers, drastically lowering the redox potential (Eh). Concomitantly, the initial irritation of the epithelium increases the flow of gingival crevicular fluid (GCF), an exudate rich in serum proteins, hemin, and transferrin. This anaerobic, alkaline, and proteinaceous microenvironment exerts absolute selective pressure that suffocates the commensal microbiota and favors the exponential proliferation of proteolytic, Gram-negative, and strictly anaerobic species, such as *Peptostreptococcus canis* and *Porphyromonas gulae*, and clusters of opportunistic pathogens such as *Pseudomonas* spp. [7]. These taxa act as keystone pathogens, orchestrating a hyperinflammatory response in the host that triggers the massive release of matrix metalloproteinases (MMPs) by neutrophils, disrupting normal bone coupling and perpetuating RANK/RANKL-mediated osteoclasia [8].

The recognition that canine periodontal disease is an environmentally driven pathology demands a reevaluation of the external macro-determinants that dictate oral ecology. In this context, dietary rheology and tribology, the science that studies the friction, wear, and deformation of food substrates during mastication, emerge as the most critical prophylactic modulators [9]. Historically, it has been documented that commercially balanced pelleted (extruded) diets have an anisotropic structural matrix. The occlusal force required to fracture these pellets induces tangential shear stress (micro-debridement) on the clinical crown, mechanically resetting the primary biofilm succession and inhibiting its geometric maturation [10]. Conversely, the contemporary shift toward soft, moist, or “homemade” diets, rich in thermogelatinized carbohydrates and highly adhesive, eliminates the coefficient of friction. This omission of basal mechanical clearance condemns the ecosystem to persistent plaque retention, irreversibly accelerating the thermodynamic “trigger” towards severe dysbiosis [11].

Alongside dietary factors, the host’s anatomical architecture determines the topography of retentive ecological niches. Classical literature has established the dogma that canine periodontal disease is almost exclusively found in small breeds or those with brachycephalic skull conformation, arguing that the shortening of the maxillomandibular axis causes rotation, dental crowding, and physiological reduction in the surrounding alveolar bone [12]. However, emerging high-resolution epidemiological analyses suggest that this view is reductionist. Recent research demonstrates that biomechanical alterations in dolichocephalic skulls, characterized by extreme elongation that generates wide diastemas and divergence in root parallelism, create “self-cleaning dead zones” [13]. In these fenestrated areas, the physiological friction exerted by the buccal mucosa and the lingual sweep are biomechanically ineffective, promoting the formation of deep and narrow periodontal pockets that operate as stable anaerobic bioreactors, with a high propensity for colonization by opportunistic Gram-negative bacilli [14].

Despite significant progress in understanding the polymicrobial and anatomical etiology of canine periodontal disease, a critical geographic and sociodemographic bias persists in the current literature. The vast majority of microbiome and epidemiological profiles originate from studies conducted in North America and Western Europe [15]. In these regions, highly specialized veterinary dentistry, annual prophylaxis under general anesthesia, and the widespread use of specific dental diets artificially modulate biofilm evolution. In contrast, in developing regions like Ecuador, the eco-epidemiological dynamic is diametrically opposed. Intense anthropomorphization of pet care practices has popularized the adoption of mixed or homemade nutritional regimens among owners who perceive “soft” as synonymous with “premium,” disregarding the biomechanical requirements of dental shear. In addition, periodontal clinical intervention in the region is usually exclusively therapeutic or rescue (exodontia) in terminal stages, rather than prophylactic [16].

This unique demographic scenario in Ecuador offers an unprecedented observational window to study the uninterrupted natural history of canine periodontal disease and the true multivariate impact of host covariates on the microbiome, free from the frequent intervention of professional debridement.

Given the background outlined above, a significant gap remains in the development of integrative analytical approaches that allow for the simultaneous evaluation of host factors and microbiological patterns in canine periodontal disease, particularly in underrepresented geographic regions such as Latin America. In this context, the objective of this study was to evaluate the association between host factors, including age, diet, and cranial morphology, and the presence and severity of canine periodontal disease in dogs treated in an urban clinical setting in Ecuador, as well as to characterize the subgingival bacterial profile recoverable by culture-based methods.

This approach allows for a comprehensive evaluation of the clinical and microbiological components of canine periodontal disease while considering the inherent limitations of culture techniques. Rather than assuming predefined hierarchical relationships between the analyzed variables, the present study aimed to quantify their relative contribution within a multivariable analytical framework, allowing for a more comprehensive understanding of the interaction between host factors and the subgingival bacterial ecosystem.

## 2. Materials and Methods

### 2.1. Study Design and Ethical Considerations

A cross-sectional, analytical, observational study was conducted to evaluate the association between host factors and the presence of canine periodontal disease in canines. The study was carried out in veterinary clinics in the urban area of Loja, Ecuador, between January and March 2025.

Participants were selected using non-probability convenience sampling, based on case availability and the operational feasibility of the participating centers. This methodological approach reflects the realities of clinical practice, although it introduces limitations in terms of population representativeness, which were considered in the interpretation of the results.

**Figure 1:**
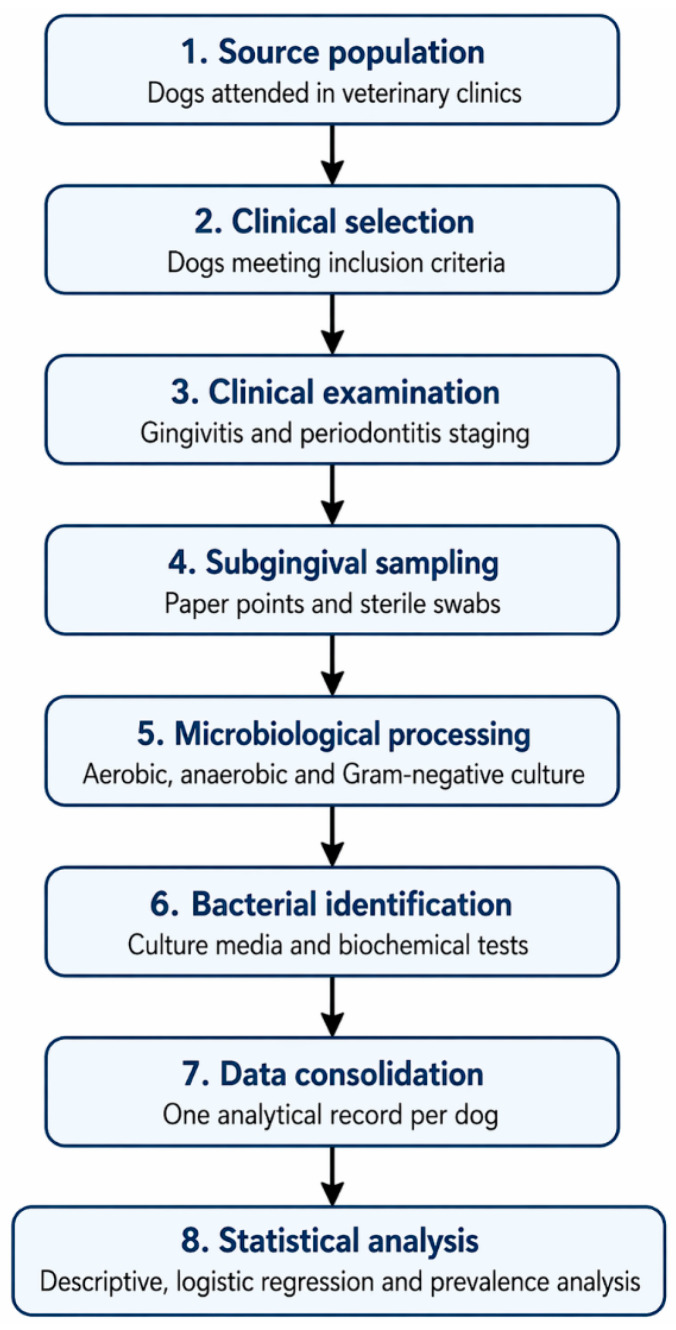
Methodological flow of the study.

The overall study design workflow comprised the stages of patient selection, periodontal clinical evaluation, subgingival sample collection, microbiological processing, and bacterial identification, as well as statistical analysis at the individual level, avoiding pseudoreplication resulting from multiple isolates per patient. These stages are summarized schematically in Figure 1 to facilitate a comprehensive understanding of the methodological procedure.

The design, analysis, and results report were structured according to the STROBE guidelines (Strengthening the Reporting of Observational Studies in Epidemiology), adapted to the context of veterinary medicine, with the aim of ensuring methodological transparency and reproducibility of the study.

The research protocol was approved by the relevant institutional ethics committee (include name, code, and date), and all clinical procedures were performed after obtaining written informed consent from the owners. Interventions were limited to routine and minimally invasive clinical procedures performed by trained veterinarians, thus ensuring the animals’ well-being throughout the process.

### 2.2. Study Population and Selection Criteria

The unit of analysis consisted of canine patients treated at veterinary clinics in the city of Loja during the study period. From a source population of 573 individuals, a sample of 100 dogs was selected using non-probability convenience sampling, based on case availability and the operational feasibility of the participating centers.

Dogs of both sexes, older than one year, with a clinical diagnosis of gingivitis or periodontitis determined by stomatological evaluation, were included. As an exclusion criterion, samples from animals that had received systemic antimicrobial treatment, anti-inflammatories, or oral antiseptics in the four weeks prior to sampling, as well as those that underwent professional dental prophylaxis in the previous six months, were discarded in order to avoid iatrogenic alterations in the oral microbiota.

Due to the study design, a control group of animals with periodontal health was not included, which limits the ability to make comparisons with eubiosis conditions and restricts the interpretation of the findings to a clinical context of disease.

The sampling approach used introduces a potential selection bias and limits the generalizability of the results to other populations, which was taken into account in the interpretation of the analyses.

### 2.3. Clinical Evaluation and Classification of Canine Periodontal Disease

Clinical evaluation of patients was performed through a general physical examination and a complete stomatological examination. Procedures were carried out under sedation or general anesthesia, at the discretion of the attending veterinarian, with basic monitoring (heart rate, respiratory rate, and oxygen saturation) to ensure patient safety and the accuracy of the periodontal examination.

Demographic and clinical variables were recorded, including age (in years), sex, diet type, and cranial morphology. Cranial morphology was classified into three categories according to standard morphological criteria described in the veterinary literature: brachycephalic, mesocephalic, and dolichocephalic.

The type of diet was determined through structured anamnesis with the owner and was classified into three categories: balanced diet (commercial dry food), homemade diet (soft preparations of domestic origin), and mixed diet (a combination of both).

Periodontal condition was assessed by visual inspection and periodontal probing of multiple teeth, considering probing depth, bleeding on probing, and clinical attachment loss. Canine periodontal disease was classified according to the American Veterinary Dental College (AVDC) criteria, categorizing individuals into stages 0 (no disease or gingivitis without attachment loss), 1 (mild periodontitis), 2 (moderate), 3 (advanced), and 4 (severe).

For statistical analysis, two outcomes were defined: (i) presence of canine periodontal disease (stages 1–4 vs. stage 0) and (ii) advanced periodontitis (stages 2–4 vs. stages 0–1).

### 2.4. Collection of Subgingival Samples

Subgingival samples were collected under standardized aseptic conditions. Prior to collection, the supragingival biofilm was removed with sterile gauze, and the operative field was isolated with absorbent material to minimize contamination.

Samples were obtained by inserting sterile paper points into the gingival sulcus or periodontal pocket. Two to four representative sites were sampled from each animal, selected based on the presence of clinical signs of disease (increased probing depth or gingival inflammation). The paper points were held in place for approximately 20 s to allow for the absorption of crevicular fluid and subgingival contents.

Additionally, a supplementary swab was performed on selected dental surfaces to improve microbiological recovery.

The samples were immediately transferred to appropriate transport media: Stuart transport medium (Oxoid Ltd., Basingstoke, UK) was used for aerobic and facultative anaerobic bacteria, whereas anaerobic transport systems (Anaerobe Systems, Morgan Hill, CA, USA) were employed for strictly anaerobic microorganisms. Transport was carried out under refrigerated conditions (4–8 °C) using insulated containers, ensuring a processing time of less than 24 h.

### 2.5. Microbiological Processing and Taxonomic Identification

Microbiological processing was performed under standardized laboratory conditions to recover aerobic, facultative, and strictly anaerobic bacteria present in the subgingival samples. Upon receipt, the samples were checked for integrity, labeling, and transport time and were subsequently cultured on different media according to the bacterial group of interest.

For the isolation of aerobic and facultative anaerobic bacteria, 5% sheep blood agar and MacConkey agar (Oxoid Ltd., Basingstoke, UK) were used, and the mixture was incubated at 37 °C for 24–48 h under aerobic conditions. Mannitol salt agar (Oxoid Ltd., Basingstoke, UK), used exclusively as a selective medium for Gram-positive cocci, was employed for the selective detection of staphylococci. For the isolation of strict anaerobic bacteria, blood agar supplemented with hemin and vitamin K was used, and the mixture was incubated under anaerobic conditions at 37 °C for 7–10 days.

Isolate identification was performed using a sequential approach that included evaluation of colonial morphology, Gram staining, and basic biochemical tests, including catalase, oxidase, and coagulase. When necessary, identification was supplemented using commercial API identification systems (bioMérieux SA, Marcy-l’Étoile, France), depending on availability and the relevance of the isolate.

In accordance with the overall study objective, the microbiological component focused on characterizing the subgingival bacterial profile recoverable by culture-based methods. In this regard, each dog was considered positive for a bacterial taxon when at least one corresponding isolate was detected, regardless of the total number of isolates obtained from that taxon in the same patient.

It is important to note that this methodological approach only allows the description of the cultivable fraction of the subgingival bacterial community, so the microbiological findings should be interpreted as a partial approximation of the periodontal bacterial ecosystem.

### 2.6. Statistical Data Analysis

Statistical analysis was performed using R statistical software version 4.3.2 (R Foundation for Statistical Computing, Vienna, Austria). Prior to the analysis, a quality control and database cleaning process was carried out. Categorical variables were described using absolute frequencies and percentages, while the age variable was summarized using the mean, standard deviation, median, and range.

Since the microbiological database included multiple isolates per individual, inferential analyses were performed at the patient level to avoid pseudoreplication bias. In this respect, each dog was considered an independent unit of analysis.

To assess the association between categorical variables and periodontal status, chi-square tests or Fisher’s exact tests were used, as appropriate. Binary logistic regression models were fitted to estimate the association between host variables (age, sex, diet, and cranial morphology) and the defined outcomes: (i) presence of canine periodontal disease and (ii) advanced periodontitis. Categorical predictors with more than two levels were analyzed using reference-category parameterization within the logistic regression models.

In the microbiological analysis, the association between the presence of bacterial taxa and periodontal status was evaluated using bivariate analysis. To control for error due to multiple comparisons, the Benjamini–Hochberg correction (False Discovery Rate, FDR) was applied. A statistical significance level of *p* < 0.05 was considered.

## 3. Results

### 3.1. Baseline Characteristics of the Studied Canine Population

The analyzed cohort consisted of canine patients with a clinical diagnosis of canine periodontal disease, whose demographic, anatomical, and dietary characteristics are presented in Table 1. A predominance of males (64.0%) was observed compared to females (36.0%). Regarding diet, a balanced diet was the most frequent (53.0%), followed by a mixed diet (29.0%) and a homemade diet (18.0%). From a morphological standpoint, the dolichocephalic biotype represented the largest proportion of the sample (71.0%), followed by mesocephalic (19.0%) and brachycephalic (10.0%), demonstrating a non-homogeneous distribution of cranial conformations in the evaluated population.

Regarding the clinical gingival condition, the most frequent category was grade 3 gingivitis (37.0%), followed by grades 1 (32.0%) and 2 (31.0%). As for the classification of periodontal attachment loss, 32.0% of the individuals were in stage 0, 31.0% in stage 1, 19.0% in stage 2, and 18.0% in stage 4. Overall, canine periodontal disease was present in 68.0% of the animals evaluated, while 37.0% presented advanced clinical forms (stages 2–4).

**Figure 2:**
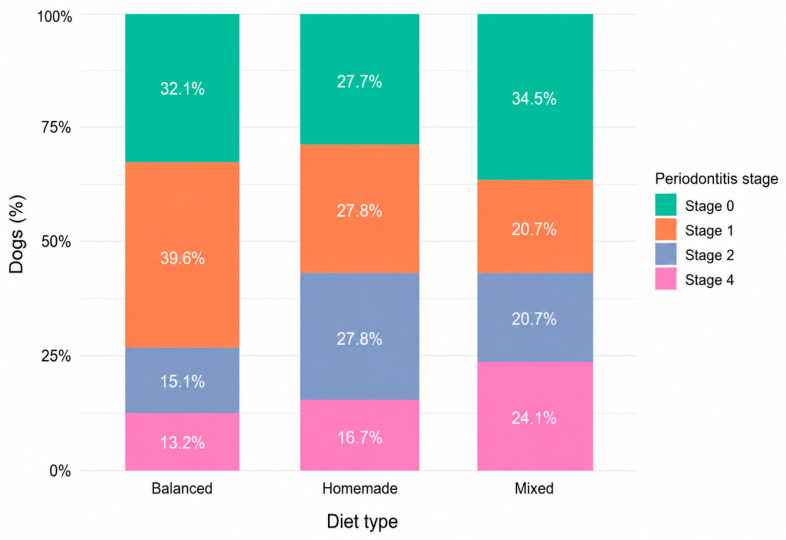
Distribution of periodontitis stages by diet type.

The relative distribution of periodontitis stages according to diet type is shown in Figure 2. In dogs fed a balanced diet, the combination of stage 1 (39.6%) and stage 0 (32.1%) predominated, while advanced stages represented smaller proportions (15.1% for stage 2 and 13.2% for stage 4). In contrast, in animals fed a homemade diet, a more homogeneous distribution among the different stages was observed, with a lower proportion of stage 0 (27.8%) and a higher relative representation of stages 2 and 4 (27.8% and 16.7%, respectively). Meanwhile, dogs on a mixed diet presented the highest relative proportion of stage 4 (24.1%), accompanied by a lower proportion of stage 1 (20.7%) compared to those on a balanced diet.

In descriptive terms, Figure 2 suggests a redistribution of the periodontal clinical profile according to dietary regimen, characterized by a lower proportion of initial forms and a greater relative representation of advanced stages in the groups with homemade and mixed diets. However, these differences should be interpreted with caution at this descriptive stage and without causal inference, since the formal evaluation of the association between diet and canine periodontal disease was subsequently carried out using multivariate models.

The age of the individuals had a mean of 4.8 ± 2.4 years, with a median of 5 years and a range of 1 to 9 years, reflecting a predominantly young to middle-aged adult cohort. In the microbiological component, the consolidated database included a total of 427 bacterial records derived from subgingival samples, corresponding to multiple isolates per individual. This represented an average of 4.27 records per dog. After removing records without bacterial growth, an average of 3.86 positive isolates per individual was obtained, demonstrating the polybacterial nature of the periodontal niche evaluated. Since the original data structure included multiple microbiological records per patient, subsequent inferential analyses were performed at the individual level to avoid biases associated with pseudoreplication and to ensure the epidemiological consistency of the results.

### 3.2. Association Between Host Factors and Presence of Periodontitis

The association between host characteristics (age, sex, diet type, and cranial morphology) and the presence of canine periodontal disease was evaluated using a binary logistic regression model, considering the presence of periodontitis (stages 1–4) as the dependent variable.

The results of the multivariable model are presented in Table 2, including the estimated odds ratios (ORs) and their respective 95% confidence intervals. In this analysis, age was identified as the only independent predictor associated with the presence of canine periodontal disease (OR = 1.18; 95% CI: 1.02–1.36; *p* = 0.021), demonstrating a progressive increase in the probability of canine periodontal disease as the individual’s age increases.

In contrast, sex did not show a significant association with the presence of canine periodontal disease (OR = 1.12; 95% CI: 0.48–2.61; *p* = 0.781). Similarly, diet type did not show significant independent associations in the adjusted model, either for the mixed diet (OR = 0.94; 95% CI: 0.39–2.24; *p* = 0.889) or for the homemade diet (OR = 1.21; 95% CI: 0.44–3.31; *p* = 0.712), compared to the balanced diet.

Regarding cranial morphology, both the mesocephalic category (OR = 1.36; 95% CI: 0.42–4.38; *p* = 0.602) and the dolichocephalic category (OR = 2.11; 95% CI: 0.68–6.54; *p* = 0.196) showed odds ratios greater than one compared to the brachycephalic category; however, these associations did not reach statistical significance in the multivariable model.

Taken together, these results indicate that, among the variables evaluated, age is the only host factor independently associated with the presence of canine periodontal disease in this cohort, while sex, diet type, and cranial morphology did not show statistically significant effects after multivariable adjustment.

### 3.3. Secondary Analysis of Advanced Periodontitis

With the aim of exploring possible factors associated with more severe forms of canine periodontal disease, an additional analysis was carried out in which the presence of advanced periodontitis, defined as stages 2–4 according to established clinical criteria, was considered as the outcome. In the initial bivariate analysis, differences in the distribution of advanced disease were observed according to some host covariates; however, these trends were re-evaluated using an adjusted multivariable logistic regression model (Table 3).

In the multivariable model, age remained a significant predictor of advanced periodontitis, showing an increased probability of severe forms as age increases (OR = 1.22; 95% CI: 1.05–1.41; *p* = 0.009). Regarding diet type, individuals fed a homemade diet showed a higher probability of developing advanced periodontitis compared to those fed a balanced diet; however, this association did not reach statistical significance in the adjusted model (*p* > 0.05). Similarly, a mixed diet also showed no significant effect. Dolichocephalic cranial morphology showed a trend toward a higher probability of advanced disease compared to brachycephalic morphology; however, this association was not statistically significant (*p* > 0.05). No significant association was observed in the mesocephalic category either. Sex showed no association with the presence of advanced periodontitis in the multivariable model.

Overall, the results indicate that, among the variables evaluated, age is the main factor associated with both the presence and severity of canine periodontal disease, while the other covariates did not show statistically significant independent effects in this cohort.

### 3.4. Subgingival Bacterial Profile in the Studied Cohort

Microbiological analysis of subgingival samples identified a diversity of bacterial taxa associated with the periodontal environment of the evaluated individuals. A total of 427 bacterial isolates were recorded, equivalent to an average of 4.27 isolates per individual. After excluding isolates without bacterial growth, an average of 3.86 positive isolates per dog was obtained, confirming the polybacterial nature of the subgingival niche in this cohort.

From a descriptive perspective, the most frequently identified bacterial taxa included *Actinomyces* spp. and *Peptostreptococcus canis*, which showed the highest detection frequencies in the cohort, followed by aerobic bacteria such as *Staphylococcus aureus* and coagulase-negative *Staphylococcus*, as well as Gram-negative bacilli such as *Pseudomonas* spp., *Klebsiella* spp., and *Escherichia coli*.

As shown in Table 4, *Actinomyces* spp. and *Peptostreptococcus canis* showed high and comparable detection frequencies between groups, with proportions close to 84% in both cases, indicating a broad distribution in the cohort regardless of periodontal status. In contrast, *Staphylococcus aureus* was detected exclusively in dogs with canine periodontal disease in this data set; however, this observation does not necessarily imply a statistically significant association, an aspect that is evaluated in subsequent inferential analyses.

For its part, coagulase-negative *Staphylococcus* showed a higher frequency in animals without disease, while Gram-negative bacilli such as *Pseudomonas* spp., *Klebsiella* spp., and *Escherichia coli* were identified in both groups with moderate differences in their relative frequencies, without showing a consistent differential pattern in this descriptive analysis.

Taken together, these results demonstrate a polymicrobial bacterial profile characterized by the coexistence of multiple taxa in the subgingival environment. However, these findings should be interpreted with caution, since the culture-based approach only allows for the identification of the culturable fraction of the bacterial community.

### 3.5. Frequency of Positive Dogs per Taxon According to Clinical Stage

To evaluate the potential association between the presence of specific bacterial taxa and periodontal status, a bivariate analysis was performed at the individual level. For each taxon, the proportion of positive dogs was compared between animals with and without canine periodontal disease using chi-square or Fisher’s exact tests, as appropriate.

The results of these analyses are presented in Table 4. Although some taxa showed apparent differences in their frequency between groups, these differences did not remain statistically significant after correction for multiple comparisons using the False Discovery Rate (FDR) method.

*Actinomyces* spp. and *Peptostreptococcus canis*, which had the highest detection frequencies in the cohort, did not show a significant association with periodontal status. Similarly, Gram-negative bacilli, including *Pseudomonas* spp., *Klebsiella* spp., and *Escherichia coli*, were identified in both groups without showing statistically significant differences.

Although some taxa, such as *Staphylococcus aureus* and coagulase-negative *Staphylococcus*, showed differential distributions between the groups in the descriptive analysis, these differences did not translate into statistically significant associations after adjustment for multiple comparisons.

Taken together, these results indicate that no single bacterial taxon was independently associated with the presence of canine periodontal disease in this cohort, supporting the polymicrobial and nonspecific nature of the subgingival bacterial ecosystem.

### 3.6. Analytical Limitations of the Culture-Based Microbiological Approach

The microbiological results presented in this study should be interpreted within the context of the inherent limitations of the methodological approach employed. In particular, bacterial identification was based exclusively on culture techniques, which restricts detection to microorganisms that are viable and cultivable under the experimental conditions used.

This approach implies a potential underrepresentation of subgingival bacterial diversity, given that a significant proportion of the microorganisms present in this ecological niche are not cultivable using conventional methods. Consequently, the bacterial profiles described reflect only a fraction of the total microbial ecosystem, thus limiting the comprehensive characterization of the periodontal microbiome.

Additionally, incubation conditions, selected culture media, and the physiological requirements of the microorganisms can introduce biases in the differential recovery of certain taxa, favoring the detection of faster-growing bacteria or those with lower metabolic requirements. This can influence both the observed relative frequencies and the interpretation of the descriptive patterns presented.

From an analytical perspective, the culture-based approach, along with the available sample size, limits the ability to detect low-magnitude associations between individual bacterial taxa and periodontal status. In this regard, although corrections for multiple comparisons were applied using the false discovery rate (FDR) method, the absence of statistically significant associations should not be interpreted as conclusive evidence of the absence of a biological relationship.

Furthermore, the cross-sectional nature of the study design prevents establishing temporal or causal relationships between the presence of certain microorganisms and the development of canine periodontal disease, so the results should be interpreted in terms of coexistence or distribution patterns rather than as direct etiological relationships.

Taken together, these limitations highlight the need to complement future studies with high-throughput molecular approaches, such as next-generation sequencing techniques, that allow for a more complete and accurate characterization of the subgingival microbiome in canines.

## 4. Discussion

This study provides clinical and microbiological evidence on canine periodontal disease in an urban cohort from Loja, Ecuador, and yields three key findings. First, canine periodontal disease showed a high clinical burden in the analyzed population, with a prevalence of 68.0% and a proportion of 37.0% of advanced forms, confirming that it is not a marginal condition but rather a frequent health problem within the spectrum of veterinary care evaluated. Second, among the host covariates examined, age was the only factor that maintained an independent and consistent association with both the presence and severity of canine periodontal disease. Third, although the microbiological analysis revealed a broad polymicrobial bacterial community, no individual taxon showed a statistically significant association with periodontal status after applying the correction for multiple comparisons, shifting the interpretation from a model focused on single pathogens to one of community coexistence and ecological imbalance.

From an epidemiological point of view, the magnitude of the disease observed in this cohort is consistent with the veterinary literature, which recognizes canine periodontal disease as one of the most frequent oral pathologies in dogs and describes a progressive increase in its frequency with age, within a multifactorial context influenced by various host attributes [17,18]. In this regard, the fact that 68 out of every 100 dogs in this study presented with periodontitis, and that 37 out of every 100 were in stages 2–4, not only reinforces the clinical relevance of the problem but also suggests a predominantly late detection of cases, consistent with scenarios in which periodontal care is more reactive than preventive. This interpretation is plausible considering that the cohort comes from urban clinics in a region where specialized dental intervention is not necessarily performed systematically or early [19].

The most robust finding of the multivariable analysis was the association between age and canine periodontal disease. The odds ratio (OR) of 1.18 for the presence of periodontitis implies that, for each additional year of age, the odds of disease increase by approximately 18%, while the OR of 1.22 for advanced periodontitis suggests an increase of nearly 22% per year in the odds of more severe forms. Translated into epidemiological terms, this should not be interpreted as a merely chronological effect, but rather as the cumulative expression of prolonged exposure to biofilm, sustained inflammation, and adverse tissue remodeling. In other words, age is likely acting as a summary variable of the biological duration of exposure to a persistently challenged oral ecosystem [20]. This interpretation is consistent with the available literature. The review by Niemiec et al. [1] highlights that age is one of the best-supported risk factors in canine periodontal disease, and the study by O’Neill et al. [21] showed that the frequency of periodontal diagnosis increases significantly with age. Therefore, the results of this study not only reproduce an expected observation but also quantify it in a Latin American cohort that is underrepresented in the literature, which adds external and regional value to the finding.

In contrast, the adjusted model did not identify independent associations with diet type. This point warrants particularly careful interpretation because the descriptive analysis did show a suggestive visual pattern: as shown in Figure 2, homemade and mixed diets exhibited a greater relative representation of stages 2 and 4 than the balanced diet. However, this descriptive signal did not hold true in the multivariable model, where the odds ratios (ORs) for the mixed and homemade diets were close to 1 and had wide confidence intervals. This decoupling between the descriptive and adjusted models is methodologically relevant. It suggests that the effect of diet, if any, could be mediated or confounded by other variables not captured by the model, such as actual food consistency, frequency of consumption, chewing behaviors, access to oral hygiene, variability within the “homemade” category, or differences in owner profiles [22]. It also suggests that the dietary classification used, while clinically reasonable, may not have captured the complexity of food exposure with sufficient resolution [23]. This interpretation is more prudent and sound than concluding that diet does not matter biologically; what the data suggest is that, in this cohort and under this classification, diet did not show a statistically significant independent effect [24]. This reading is consistent with the contemporary literature, in which some studies find differences in the oral microbiome between wet and dry diets, but these differences do not necessarily translate linearly into an independent clinical risk of canine periodontal disease [25].

Regarding cranial morphology, the results of this study did not show statistically significant associations between the presence or severity of canine periodontal disease in the adjusted models, although the dolichocephalic category showed odds ratio estimates greater than one. These findings suggest an inconclusive trend, insufficient to establish an independent association in the analytical context used. Additionally, the relatively limited representation of brachycephalic dogs in the study population may have reduced the statistical power to detect morphology-related differences, and therefore, these findings should be interpreted cautiously. Recent evidence indicates that cranial morphology can influence susceptibility to canine periodontal disease through anatomical factors such as dental crowding, occlusion, and dental arch conformation; however, its effect is modulated by additional host and environmental variables, including age, body size, oral hygiene, and dietary management [26]. In this sense, the results of this study are consistent with a multifactorial model, in which cranial morphology does not act as an independent predictor but rather as a potential component within a system of more complex biological and clinical interactions.

From a microbiological perspective, the findings are particularly relevant for two reasons. First, the subgingival environment was clearly polymicrobial: 427 bacterial records in 100 individuals, with an average of 3.86 positive isolates per dog after removing records without growth. Second, the most frequent taxa, *Actinomyces* spp. and *Peptostreptococcus canis*, were distributed very similarly between animals with and without canine periodontal disease, around 84% in both groups, while other taxa with visually striking patterns, such as *Staphylococcus aureus* or coagulase-negative *Staphylococcus*, lost significance after applying the False Discovery Rate (FDR) adjustment. This is relevant because it shifts the interpretation from searching for a “culprit” bacterium to evaluating a shared bacterial system whose pathological relevance likely depends more on community structure, relative abundance, metabolic interaction, and inflammatory context than on the mere presence or absence of isolated species [27].

This pattern fits well with the contemporary conceptual framework of polymicrobial dysbiosis. Hajishengallis et al. [28] have proposed that, in periodontitis, microbial communities act as quasi-organismic entities in which pathological properties emerge from cooperative interactions and altered host-microbiota dialogue rather than from a single dominant pathogen. Although this framework has been developed primarily from human periodontology, its ecological logic is relevant here: the fact that *Actinomyces* spp. and *P. canis* are very frequent in both groups suggests that their simple detection does not discriminate between disease states and that the truly informative component probably lies in community organization, the local microenvironment, and the host response. Along the same lines, studies by Li et al. [29] in dogs with healthy gingiva, gingivitis, and mild periodontitis have shown that bacterial profiles vary according to clinical status, but also that many bacteria are widely distributed across different categories, making a single-species interpretation of the process difficult. The findings of the present study are consistent with this interpretation: a broad community, with the coexistence of Gram-positive, Gram-negative, and recoverable bacteria under different conditions, without solid evidence to elevate a single taxon to the category of an independent disease marker.

In the case of *Staphylococcus*, the descriptive analysis of *S. aureus* showed its presence exclusively in dogs with canine periodontal disease; however, this signal did not persist after inferential analysis with correction for multiple comparisons, preventing the establishment of an independent association with periodontal status. This result is consistent with evidence indicating that numerous taxa can be detected in both healthy and diseased conditions and that their mere presence is not sufficient to infer a direct etiological role [30]. In this context, the detection of *S. aureus* should be interpreted with caution and as a descriptive finding that could reflect the complexity of the subgingival ecosystem, rather than as a specific disease marker; it requires validation in studies with larger sample sizes or using higher-resolution molecular approaches [31].

A key methodological aspect is the explicit distinction between descriptive and inferential analysis in the evaluation of microbiological data. Characterizing frequencies by taxon and periodontal status allows for the identification of distribution patterns, while inferential analysis, adjusted for multiple comparisons, assesses the statistical consistency of these differences. This distinction is fundamental in studies with multiple taxa, since variations observed at the descriptive level do not necessarily translate into statistically significant associations. In this context, the application of error control methods such as the False Discovery Rate (FDR) is a recommended practice to reduce the probability of false positives in multivariate or high-dimensional analyses [32]. This approach is particularly relevant in microbiological studies, where the complexity of bacterial communities and the large number of comparisons can lead to misinterpretations if appropriate corrections are not applied [33]. Consequently, the integration of descriptive and inferential analyses with FDR control strengthens the validity of the findings and allows for a more rigorous interpretation of the relationship between subgingival microbiota and periodontal status.

From a methodological perspective, the microbiological results must be interpreted within the limitations inherent to culture-based approaches. Recent evidence in dogs indicates that a substantial proportion of oral microbial diversity is not recoverable using conventional techniques and that the structure and dynamics of the subgingival biofilm are more accurately characterized by high-resolution molecular and metagenomic approaches [34]. In this regard, sequencing-based studies have demonstrated that canine oral bacterial communities exhibit high taxonomic and functional complexity, with numerous non-culturable microorganisms contributing to the ecological organization of the system [35]. Therefore, the results of this study should be interpreted as a characterization of the culturable fraction of the subgingival microbiota under specific experimental conditions. This consideration is particularly relevant, as the limitations of culture can restrict the detection of differential associations between taxa and clinical states, not necessarily due to an absence of biological differences, but rather due to the partial resolution of the method in the face of a broader and more complex microbial community [36]. Taken together, these elements reinforce the need to complement classical approaches with molecular methodologies in future studies to achieve a more comprehensive characterization of the canine oral microbiota and its relationship to canine periodontal disease.

The present findings may also be contextualized in relation to sequencing-based microbiome studies, which have become increasingly common in periodontal research. High-throughput molecular approaches have demonstrated that the canine subgingival microbiota is considerably more diverse than that recoverable through conventional culture methods, particularly regarding fastidious anaerobic taxa. Nevertheless, several bacterial groups identified in the present study, including *Actinomyces* spp., *Peptostreptococcus* spp., and *Staphylococcus* spp., have also been consistently reported in sequencing-based investigations, suggesting that culture-dependent methods may still capture clinically relevant components of the canine periodontal microbiota despite their lower taxonomic resolution.

It is also important to emphasize that the cross-sectional design limits the ability to establish temporal relationships between bacterial colonization and the development of canine periodontal disease. This type of approach allows for the identification of patterns of coexistence, distribution, and statistical association between taxa and clinical states, but it does not allow us to determine whether a microorganism precedes the onset of disease, emerges as a consequence of changes in the periodontal microenvironment, or simply coexists in both scenarios. This limitation has been widely recognized in studies of the canine oral microbiome, where it has been demonstrated that multiple taxa are present in both healthy and diseased conditions, hindering the inference of direct causal relationships [37]. In this context, the presence of shared bacteria between groups should be interpreted with caution, since a taxon frequently found in pathological states does not necessarily act as the primary etiological agent but may represent an opportunistic organism adapted to an altered ecological niche. This pattern is consistent with contemporary ecological models applied to the oral microbiome in dogs, where dysbiosis is understood as a reorganization of the microbial community rather than the action of a single dominant pathogen [38].

In clinical terms, the study results have significant implications. Recent evidence in canine populations indicates that canine periodontal disease has a high prevalence and a progressive nature associated with age, reinforcing the need for early preventive strategies based on sustained biofilm control and regular oral cavity assessment [39]. In this regard, contemporary clinical guidelines emphasize the implementation of preventive dental care programs and regular monitoring as central elements in oral health management in dogs [40]. Additionally, evidence suggests that periodontal risk cannot be attributed solely to individual variables but rather results from the interaction of multiple host and environmental factors, including age, clinical management, and oral microenvironment conditions [41].

Among the study’s strengths are the integration of clinical, anatomical, and bacteriological variables within a single analytical framework and the consolidation of the analysis at the individual level, which avoids biases arising from pseudoreplication in microbiological studies. Furthermore, the use of multivariable models and the application of False Discovery Rate (FDR) adjustments represent best practices in analyses with multiple comparisons, as they improve inferential robustness and reduce the likelihood of spurious results [42].

Additionally, sample size can affect the detection of low-magnitude associations, and the culture-based approach underestimates true bacterial diversity, given that a significant proportion of the oral microbiome is not recoverable using conventional techniques [43]. These limitations are consistent with recent studies of the canine oral microbiome, which highlight the need for molecular approaches for more comprehensive characterization.

Taken together, the results position canine periodontal disease as a frequent, progressive, and age-dependent process in which the microbiological signal at the culture level is insufficient to independently discriminate clinical states. This evidence supports an interpretive approach based on microbiome ecology and host–environment interactions, rather than on the identification of individual pathogens [44]. From this perspective, future research should prioritize longitudinal designs, the inclusion of healthy controls, and the use of high-resolution molecular tools to advance toward a more comprehensive characterization of subgingival dynamics in dogs.

## 5. Conclusions

In this clinical cohort, canine periodontal disease showed a high prevalence, with 68% of dogs affected and 37% presenting moderate to advanced stages, confirming its clinical relevance in urban settings. Multivariable analysis identified age as the most consistent risk factor, with a significant increase in the probability of disease with increasing age, whereas variables such as cranial morphology and diet did not show independent associations after adjustment. From a microbiological perspective, the results revealed a broad bacterial community shared across clinical stages, with no culturable taxa acting as independent markers of disease after FDR correction, supporting a polymicrobial and ecological interpretation of the process. Taken together, these findings suggest that canine periodontal disease in dogs follows a multifactorial and cumulative model, in which age and host context have a greater impact than isolated variables or individual taxa. These results reinforce the need for early preventive strategies and future studies that integrate larger sample sizes and high-resolution molecular approaches to better understand the dynamics of the subgingival microbiome.

## Figures and Tables

**Table 1:** Demographic, clinical, and anatomical characteristics of the evaluated canine cohort.

Variable	Category	*n*	%
Age (years)	Mean ± SD	4.8 ± 2.4	—
	Median (range)	5 (1–9)	—
Sex	Female	36	36.0
	Male	64	64.0
Diet	Balanced	53	53.0
	Mixed	29	29.0
	Homemade	18	18.0
Cranial morphology	Brachycephalic	10	10.0
	Mesocephalic	19	19.0
	Dolichocephalic	71	71.0
Gingivitis	Grade 1	32	32.0
	Grade 2	31	31.0
	Grade 3	37	37.0
Periodontitis	Stadium 0	32	32.0
	Stadium 1	31	31.0

Note: The periodontal clinical classification was performed according to the criteria of the American Veterinary Dental College (AVDC).

**Table 2:** Association between host factors and presence of periodontitis (multivariable logistic regression model).

Variable	Category	OR	95% CI	*p*-Value
Age (years)	Continuous	1.18	1.02–1.36	0.021
Sex	Male vs. Female	1.12	0.48–2.61	0.781
Diet	Mixed vs. Balanced	0.94	0.39–2.24	0.889
	Homemade vs. Balanced	1.21	0.44–3.31	0.712
Cranial morphology	Mesocephalic vs. Brachycephalic	1.36	0.42–4.38	0.602
	Dolichocephalic vs. Brachycephalic	2.11	0.68–6.54	0.196

Note: OR: odds ratio; 95% CI: 95% confidence interval. Balanced diet and brachycephalic cranial morphology were used as reference categories. Therefore, pairwise contrasts not explicitly shown in the table (e.g., mixed vs. homemade diet or mesocephalic vs. dolichocephalic morphology) can be inferred indirectly from the reported model coefficients.

**Table 3:** Association between host factors and advanced periodontitis (multivariable logistic regression model).

Variable	Category	OR	95% CI	*p*-Value
Age (years)	Continuous	1.22	1.05–1.41	0.009
Sex	Male vs. Female	1.08	0.46–2.54	0.852
Diet	Mixed vs. Balanced	1.12	0.46–2.74	0.802
	Homemade vs. Balanced	1.74	0.62–4.89	0.294
Cranial morphology	Mesocephalic vs. Brachycephalic	1.48	0.44–4.96	0.531
	Dolichocephalic vs. Brachycephalic	2.36	0.73–7.61	0.149

Note: OR: odds ratio; 95% CI: 95% confidence interval. Balanced diet and brachycephalic cranial morphology were used as reference categories. Therefore, pairwise contrasts not explicitly shown in the table (e.g., mixed vs. homemade diet or mesocephalic vs. dolichocephalic morphology) can be inferred indirectly from the reported model coefficients.

**Table 4:** Association between bacterial taxa and periodontal status in canines.

Taxon	No Disease (%)	Disease (%)	*p*-Value	FDR-Adjusted *p*
*Actinomyces* spp.	84.4	83.8	0.94	0.99
*Peptostreptococcus canis*	84.4	83.8	0.94	0.99
Coagulase-negative *Staphylococcus*	87.5	29.4	<0.001	0.06
*Staphylococcus aureus*	0.0	66.2	<0.001	0.07
*Pseudomonas* spp.	28.1	26.5	0.85	0.95
*Klebsiella* spp.	28.1	20.6	0.41	0.88
*Citrobacter* spp.	18.8	11.8	0.36	0.84
*Enterobacter* spp.	3.1	13.2	0.08	0.40
*Salmonella* spp.	15.6	5.9	0.12	0.48
*Micrococcus* spp.	12.5	2.9	0.11	0.47
*Escherichia coli*	3.1	4.4	0.72	0.95

Note: Percentages were calculated at the individual level. Each dog was considered positive when at least one isolate of the corresponding taxon was detected. *p*-values were obtained using chi-square or Fisher’s exact tests. A correction for multiple comparisons was applied using the Benjamini–Hochberg (FDR) method.

## Data Availability

The original contributions presented in this study are included in the article. Further inquiries can be directed to the corresponding author.
